# Effect of Mechanical Stresses in Rapidly Heated Fe_73_Cu_1_Nb_3_Si_16_B_7_ Ribbon Arising During the Ring Core Formation on Their Magnetic Properties

**DOI:** 10.1186/s11671-017-2041-9

**Published:** 2017-04-26

**Authors:** Anton Nosenko, Taras Mika, Olexandr Semyrga, Viktor Nosenko

**Affiliations:** 0000 0004 0385 8977 grid.418751.e G.V. Kurdyumov Institute for Metal Physics of National Academy of Sciences of Ukraine, 36, Academician Vernadsky Boulevard, Kyiv, 03142 Ukraine

**Keywords:** Nanocrystalline magnetic core, Induced magnetic anisotropy, Tensile stress, Winding-induced mechanical stresses

## Abstract

The influence of winding-induced mechanical stresses on the magnetic anisotropy and core loss in toroidal cores made of Fe_73_Cu_1_Nb_3_Si_16_B_7_ ribbon is studied. The ribbon for the cores was rapidly pre-heated under tensile stress up to 120 MPa. It was found that magnetic characteristics of the material (magnetic anisotropy energy and the core loss) can be controlled by varying the tensile stress during the preliminary rapid heating of the ribbon. It was shown that with reducing core diameter, the magnetic anisotropy energy and core loss significantly increase. However, relatively high winding-induced core loss in small cores can be significantly reduced by increasing tensile stresses applied to the ribbon during pre-heating.

## Background

Nowadays, soft magnetic nanocrystalline alloys of Fe–Nb–Cu–B–Si system [1] are widely used in magnetic cores of various inductive components (transformers or chokes). It is known that formation of α–Fe(Si) nanocrystals in these alloys during heat treatment improves their soft magnetic properties. Volume fraction of nanocrystals in these materials is 75–80% and their size is about 10 nm [1]. The so-called linear hysteresis loop can be obtained in these types of alloys by inducing uniaxial transverse magnetic anisotropy during annealing under tensile stress [2, 3]. Magnetic structure of such type alloys was studied in details by the authors of [4]. In [5], huge magnetic anisotropy was reported for the ribbons of this type.

Magnetic cores made of the ribbons with induced magnetic anisotropy have a number of advantages, namely, high field stability of magnetic permeability [6], low core loss in the important frequency range (1–100 kHz) [6], and DC bias immunity [7].

The disadvantage of these cores is the sensitivity of the magnetic properties of the ribbon to mechanical stresses arising during core formation [8, 9]. Difficulties arise during production of miniaturized magnetic nanocrystalline cores with transverse anisotropy, particularly for pulse transformers in telecommunication systems. That is why the purpose of the current investigation was to study the influence of mechanical stresses appearing during core fabrication on the transverse magnetic anisotropy that was induced by tensile stress during nanocrystallization process and core loss.

## Methods

Fe_73_Nb_3_Cu_1_B_7_Si_16_ amorphous ribbon (the thickness 20 μm, width 10 mm) was obtained by planar flow casting process (PFC) in the air atmosphere using the equipment for rapid quenching of the melt [10].

Straight pieces of ribbons were heated in order to obtain nanocrystalline structure of the material. Fast heating was realized by conducting of electric current with the density *j*_*h*_ = 42 A/mm^2^ and frequency 50 Hz through straight piece of ribbon for *t*_*h*_ = 3.7 s that provided its heating above 600 °C. To induce a uniaxial transverse magnetic anisotropy in the ribbons, the rapid heating is done under tensile stress *σ*_*t*_ = 0, 35, 80, and 120 MPa.

**Fig. 1:**
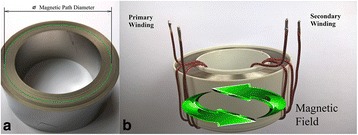
The core made of the ribbon heated by electric current under tensile stresses (**a**) and the same core in a plastic box with primary and secondary winding (**b**)

**Fig. 2:**
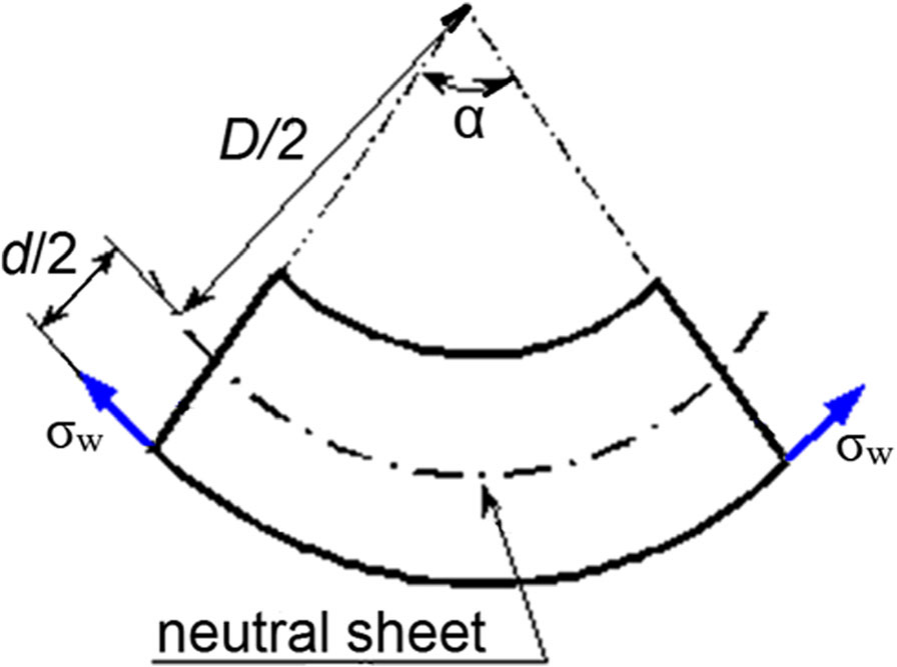
Schematic segment of the bended ribbon in the core

After the heating, the ribbon was used to produce cores with different magnetic path diameters *D* ≈ 5÷70 mm (Fig. 1a). Reducing the core diameter caused increasing of winding-induced mechanical stresses in the deformed ribbon. Winding-induced mechanical stresses *σ*_*w*_ were calculated by Hooke’s law for normal stress during straight bending:

1 $$ {\sigma}_w= E\cdot \varepsilon = E\cdot \frac{\left(\frac{D}{2}+\frac{d}{2}\right)\cdot \alpha -\frac{D}{2}\cdot \alpha}{\frac{D}{2}\cdot \alpha}= E\cdot d/ D $$

where *E* is Young’s modulus, *ε* is the deformation, *d* is the ribbon thickness (20 μm), *D* is the magnetic path diameter, and *α* is the angle of the ribbon segment (Fig. 2).

Mechanical testing of ribbons had been done using the universal servohydraulic machine Instron 8802. Loading curves of ribbons during tests on uniaxial tensile stretch were recorded in coordinates “stress-strain” that allowed to determine Young’s modulus.

Magnetic studies were done using toroidal (ring) cores with primary and secondary windings like in a usual transformer (i.e., the magnetic field was applied along the ribbon axis (Fig. 1b)). Dynamic B-H curves and core loss at different frequencies were measured using the measuring complex MS-02 B-H ANALYZER (MSTATOR, Novgorodskaya oblast, Russia). The detailed description of this complex can be found in [6].

## Results and Discussion

The technological processes used for the fabrication of the cores studied in the present paper imply application of two types of mechanical stresses into the material. One type is the tensile stress which is applied during the pre-heating of amorphous ribbons. As it was shown in [6], this procedure causes irreversible changes in the inner structure of amorphous ribbons on the nanoscale level. Another type of stress is the winding-induced bending of the ribbons. This stress is elastic and permits multiple winding-unwinding cycles. The interplay of these stress influences provides the possibilities to tune the magnetic properties of the material and achieves the targeted parameters of the cores.

**Fig. 3:**
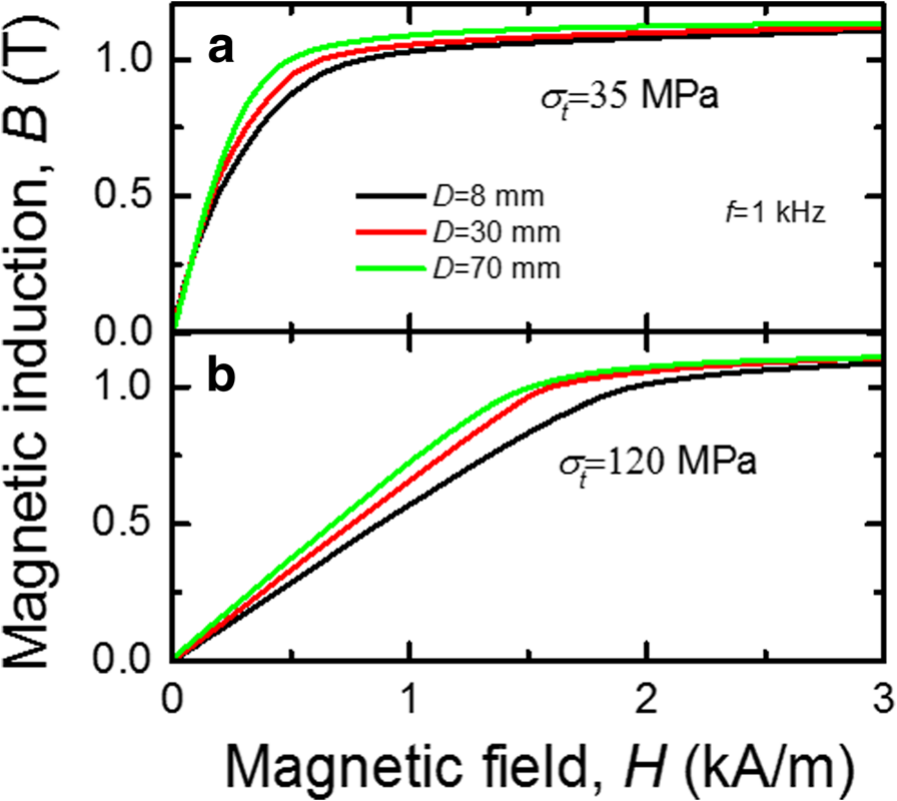
Remagnetization loop of the cores made of the ribbons pre-heated by electric current under the tensile stress 35 MPa (**a**) and 120 MPa (**b**)

Figure 3 shows the remagnetization loops of three cores with different diameters. These cores were fabricated using the ribbon pre-treated by rapid heating under various tensile stresses of 35 and 120 MPa at the frequency of *f* = 1 kHz. Figure 3a shows that the reduction of the magnetic path diameter *D* leads to the rounding of remagnetization loops of the cores made of the ribbons pre-heated under the tensile stress 35 MPa. This behavior is caused by the increased winding-induced mechanical stresses in the ribbon. Cores made of the ribbons pre-heated at the tensile stress of 120 MPa are sensitive to winding-induced stresses too (Fig. 3b), but these cores demonstrate linear loops regardless the core diameter.

The energy of uniaxial transverse magnetic induced anisotropy *K* in the core formed using the ribbon pre-heated under tensile stress is equal to the area above the remagnetization loop curve (Fig. 3) and can be obtained by numerical integration

2 $$ K={\displaystyle \underset{0}{\overset{1.15}{\int }} H\cdot dB} $$

**Fig. 4:**
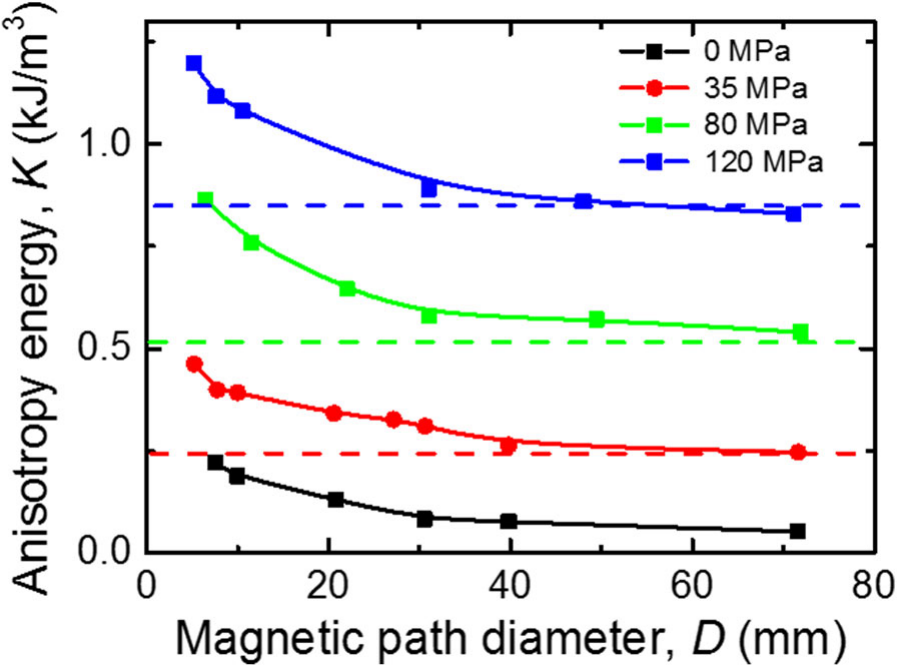
Winding-induced uniaxial magnetic anisotropy energy *K* vs. magnetic path diameter

Figure 4 shows the induced magnetic anisotropy energy *K* vs. magnetic path diameter *D* for the cores made of the ribbon rapidly pre-heated under tensile stress *σ*_*t*_. The induced magnetic anisotropy increases with the winding-induced mechanical stress independently from the magnetic anisotropy that was previously formed during the pre-heating under tensile stress. Values of the induced magnetic anisotropy in similar ribbons studied in [11] without winding-induced mechanical stress are shown as dotted line in Fig. 4 for comparison. It is seen that the magnetic anisotropy of large diameter cores almost coincides with the anisotropy obtained for the straight ribbons in [11]. It is also seen that the influence of the core diameter on the induced magnetic anisotropy energy *K* is more pronounced when *D*_*c*_ ≤ 30 mm in all cores. Probably, the bending stresses in the cores of small diameters are sufficiently high to observe the contribution of magnetoelastic effect. The magnetic anisotropy energy *K*_*im*_ caused by winding-induced stress *σ*_*w*_ is:

3 $$ {K}_{im}=3/2\cdot {\lambda}_s\cdot {\sigma}_w, $$

where *λ*_*s*_ is the saturation magnetostriction.

**Fig. 5:**
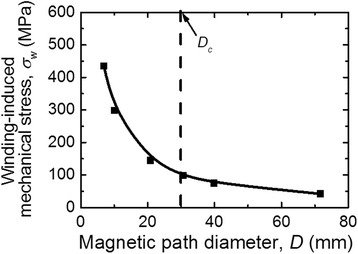
Dependence of winding-induced mechanical stress vs. magnetic path diameter

Figure 5 shows the dependence of winding-induced mechanical stress on the core diameter *D* calculated using the formula (1). It is seen that at *D* ≤ *D*_*c*_, the winding-induced mechanical stress steeply increases which corresponds to the increase of the induced magnetic anisotropy energy *K* (at *D* ≤ *D*_*c*_) in Fig. 4.

Substituting (1) into (3) and taking into account the additional anisotropy *K*_*is*_ which is induced in the unloaded ribbon during the pre-heating in air [12], we get the formula for calculating the magnetic anisotropy energy at zero creep (*σ*_*t*_ = 0 MPa)

4 $$ K=3/2\cdot {\lambda}_s\cdot E\cdot d/ D+{K}_{is}. $$

The origin of the additional magnetic anisotropy *K*_*is*_, according to [12], is the influence of the environment, i.e., the interaction of ribbon’s surfaces with oxygen, hydrogen, and water vapor which leads to anisotropic crystallization of surfaces.

**Fig. 6:**
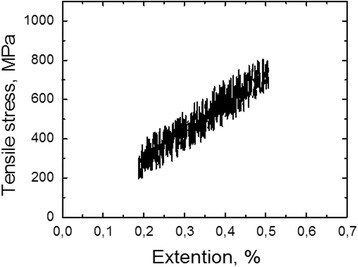
Dependence of relative elongation of nanocrystalline ribbon (pre-heated under the tensile stress 80 MPa) on tensile stress

Young’s modulus of rapidly heated ribbons at various tensile stresses was calculated by Hooke’s law using the load-deformation curves (Fig. 6). The value of *E* ≈ 150 GPa is smaller than the one obtained in [13] (*E* ≈ 190 GPa) for such type of alloys. Perhaps this discrepancy is caused by the differences in the methods of measurements and chemical compositions of alloys.

Using (4) for the fitting of the experimental results for the cores made of the ribbon pre-heated at zero tensile stress (Fig. 4, curve 0 MPa), we obtained the values *λ*_*s*_ = 0.31 × 10^−6^ and *K*_*is*_ = 0.042 kJ/m^−3^. The *λ*_*s*_ value correlates with the one obtained in [14].

**Fig. 7:**
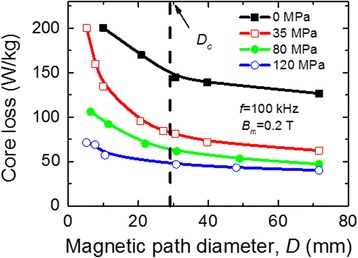
Dependence of core loss on their diameter

Figure 7 shows the dependence of core loss on magnetic path diameter at maximum magnetic induction *B*_*m*_ = 0.2 T and frequency *f* = 100 kHz. One can see that core loss at large core diameters (*D* > 70 mm) is approximately constant regardless of the value of the tensile stress *σ*_*t*_ applied during the pre-heating. At small core diameters (*D* < 70 mm), core loss increases with the growth of winding-induced bending stress. In order to avoid this deterioration, *D* is required to exceed a critical diameter *D*_*c*_ under which the easy axis of magnetization lies in the transverse direction in the whole core [8].

Only the cores made of the ribbons pre-heated under large tensile stress (120 MPa) show excellent stability of core loss (independence of winding-induced mechanical stress). The resulting effect can be explained by decreasing of saturation magnetostriction with increasing value of tensile stress [14].

## Conclusions

1. It is shown that the magnetic anisotropy of nanocrystalline ribbon of Fe_73_Cu_1_Nb_3_Si_16_B_7_ alloy in the toroidal core can be controlled by increasing of tensile stress applied during its preliminary rapid heating.
2. It is demonstrated that increasing of winding-induced mechanical bending stress (with decreasing of magnetic path diameter) above a certain value leads to higher magnetic anisotropy energy as well as higher core loss.
3. It is shown that the increase of core loss (due to winding-induced stress) can be significantly reduced by increasing the tensile stress applied during pre-heating of the ribbon.

Based on the results on the influence of tensile stresses during rapid heating of ribbons, the values of stresses necessary to minimize core remagnetization loss were obtained; they are important for manufacturing technology of small nanocrystalline magnetic cores with linear DC bias immune remagnetization loop, in particular for the production of pulse transformers broadband telecommunication systems.
